# Selective Pressure of Temperature on Competition and Cross-Feeding within Denitrifying and Fermentative Microbial Communities

**DOI:** 10.3389/fmicb.2015.01461

**Published:** 2016-01-07

**Authors:** Anna Hanke, Jasmine Berg, Theresa Hargesheimer, Halina E. Tegetmeyer, Christine E. Sharp, Marc Strous

**Affiliations:** ^1^Microbial Fitness Group, Max Planck Institute for Marine MicrobiologyBremen, Germany; ^2^Center for Biotechnology, Institute for Genome Research and Systems Biology, University of BielefeldBielefeld, Germany; ^3^Energy Bioengineering Group, Department of Geoscience, University of CalgaryCalgary, AB, Canada

**Keywords:** metagenomics, chemostat, marine sediment, *Rhodobacteraceae*, *Vibrio*, *Arcobacter*, microbial ecology, nitric oxide dismutase

## Abstract

In coastal marine sediments, denitrification and fermentation are important processes in the anaerobic decomposition of organic matter. Microbial communities performing these two processes were enriched from tidal marine sediments in replicated, long term chemostat incubations at 10 and 25°C. Whereas denitrification rates at 25°C were more or less stable over time, at 10°C denitrification activity was unstable and could only be sustained either by repeatedly increasing the amount of carbon substrates provided or by repeatedly decreasing the dilution rate. Metagenomic and transcriptomic sequencing was performed at different time points and provisional whole genome sequences (WGS) and gene activities of abundant populations were compared across incubations. These analyses suggested that a temperature of 10°C selected for populations related to Vibrionales/Photobacterium that contributed to both fermentation (via pyruvate/formate lyase) and nitrous oxide reduction. At 25°C, denitrifying populations affiliated with *Rhodobacteraceae* were more abundant. The latter performed complete denitrification, and may have used carbon substrates produced by fermentative populations (cross-feeding). Overall, our results suggest that a mixture of competition—for substrates between fermentative and denitrifying populations, and for electrons between both pathways active within a single population –, and cross feeding—between fermentative and denitrifying populations—controlled the overall rate of denitrification. Temperature was shown to have a strong selective effect, not only on the populations performing either process, but also on the nature of their ecological interactions. Future research will show whether these results can be extrapolated to the natural environment.

## Introduction

Denitrification is the microbial respiration of nitrate and/or nitrite with dinitrogen gas as the main end product (Kraft et al., 2011). In freshwater and coastal marine habitats, denitrification is also the main sink for nitrate originating from the agricultural use of synthetic fertilizers. Such habitats are exposed to diurnal and seasonal temperature fluctuations. In parallel, average annual temperatures slowly increase because of global warming, up to ~6°C during this century (Cubasch et al., 2013). Previous research suggested that higher temperatures stimulate denitrification relative to aerobic respiration because oxygen solubility decreases with temperature (Veraart et al., 2011). Known denitrifying bacteria are facultative aerobes so these two forms of respiration directly compete for electrons within the same respiratory chain (Chen and Strous, 2013). For this reason, a direct effect of oxygen on denitrification rates can be expected. Data from ice cores show that historically, temperature was positively correlated with atmospheric N_2_O concentration, which could be explained by a positive relationship between denitrification and temperature. Yet, in natural ecosystems, temperature may act on denitrification in more complex ways. For example, reduced oxygen solubility may also reduce nitrification rates, indirectly constraining rates of denitrification, by reducing nitrite and nitrate availability.

On average, denitrification converts a small percentage (1–5%) of the nitrate to nitrous oxide, a powerful greenhouse gas (Fowler et al., 2013). Therefore, denitrification potentially acts as a positive feedback loop on global warming, with higher temperatures stimulating denitrification, which in turn yields more nitrous oxide, leading to further warming. Reduced nitrous oxide solubility at increasing temperature, may lead to higher net emissions to the atmosphere. For these reasons, a better understanding of the relationship between temperature and denitrification is urgently needed. Unfortunately, only few studies have so far addressed this relationship (Canion et al., 2014).

Denitrification rates are not only affected by oxygen and nitrate availability, but also by the availability of energy and carbon sources. Denitrifying bacteria are generally considered chemo-organo-heterotrophs, but many chemo-litotrophic denitrifiers, that use sulfide or hydrogen as the electron donor, are also well known. Overall, heterotrophic denitrifiers are known to use a wide spectrum of carbon sources, such as short-chain fatty acids (e.g., acetate), sugars and aminoacids. Denitrifying members of the Bacteroidetes may even use macromolecules such as proteins and polysaccharides as their substrate. Therefore, in natural habitats, both competition and cross-feeding between fermentative and denitrifying bacteria can be expected.

The present study addresses the potential ecological interactions between denitrifying and fermentative bacteria, as a function of temperature. Natural microbial communities from a tidal flat in the Wadden Sea were incubated for at least 100 days in anoxic laboratory chemostats at two different temperatures (10 and 25°C). These were provided with nitrite and nitrate, as well as a mixture of organic substrates including glucose, seven different aminoacids and acetate. The composition of the organic substrates reflected the composition of decaying biomass, the main carbon and energy source in marine sediments, at least in terms of its monomers. The supplied organic substrates enabled different fermentative metabolisms (e.g., glucose vs. aminoacids) as well as anaerobic respiration in the form of denitrification (with acetate, glucose, and/or aminoacids). According to the typical model of the microbial redox tower (Fenchel and Jørgensen, 1977), most probably an over-simplification but still widely used for lack of more detailed information, denitrifying bacteria typically oxidize all these substrates directly, without the need for syntrophic partners. Thus, denitrification is assumed to differ from sulfate reduction, which typically requires substrates such as lactate, hydrogen, acetate and butyrate, provided by fermentative bacteria (Plugge et al., 2011). Simultaneous supply of all these different organic compounds enabled us to probe to what extent denitrifying and fermentative bacteria would compete for substrates or engage in syntrophic partnerships. Alternatively, there was also the possibility that fermentation and denitrification would be performed in parallel by a single population. A mixed fermentative/respiratory metabolism is well known for many aerobic enteric bacteria (e.g., *E. coli*), baker's yeast and sulfate reducing bacteria (Plugge et al., 2011).

Overall conversions of the substrates were determined and the enriched microbial communities were analyzed by shotgun sequencing of extracted DNA and RNA in a genome-centric approach. Assembly and binning yielded provisional whole genome sequences (WGS) for most of the abundant community members. All genomes were annotated and per-gene transcriptional activities were determined for each population by mapping transcriptomic reads. This way, it was possible to assess which type of ecological interactions between denitrifying and fermentative populations occurred and whether this was affected by temperature.

Thus, in its experimental design the present study aims to bridge the gap between conventional microbial physiology and the direct study of natural ecosystems. Whereas the former enables non-ambiguous conclusions about highly simplified systems (typically a single isolate or a single substrate), the latter addresses the entire ecosystem in full complexity, but generally does not resolve causal relationships very well. Investigation of a simplified, but still complex, ecosystem, enabled by genome-centric metagenomics, provided new insight in the ecological interaction between denitrifying and fermentative populations and provided meaningful questions for future study. One may still argue that the laboratory chemostat is only remotely similar to the natural ecosystem: hydrolysis of macromolecular substrates was not addressed and spatio-temporal dynamics were different from the natural ecosystem (suspended bacteria vs. a dynamic, spatially structured sediment). However, use of chemostats did enable growth at low, micromolar range, substrate concentrations and more or less stable conditions for long periods of time. This at least approximated the natural ecosystem much better than would have been possible in even more simplified conventional microbial physiology studies. The results suggest that temperature affects the nature of the ecological interactions, resulting in a stimulation of denitrification that goes beyond simple predictions based on oxygen solubility or denitrification kinetics measured for isolated strains of denitrifying bacteria.

## Materials and methods

### Sampling and inoculation

Sediment samples were taken from the intertidal flat “Janssand” located in the Central German Wadden Sea south of the Eastern Friesian Island Spiekeroog. Sampling was conducted twice in subsequent years to receive inocula for two replicate chemostat experiments. First on March 9th 2011 (N: 053° 44′ 153″/E: 007° 41′ 943″, 3°C, overcast), second on March 6th 2012 (N: 053° 44′ 133″/E: 007° 41′ 989″, 9°C, sunny). Sampling procedure and preparation was according to Hanke et al. (2014). Briefly, sediment from the upper 2 cm layer of Janssand was shoveled into a cooling box with a trowel, and kept on ice during 5 h transport to the lab, where it was diluted with artificially prepared seawater in a ratio of 1:1 and mixed for 3 min with a cement mixer. The turbid, sand free supernatant (the “cell extract”) was filled into 1 l transfusion bottles and made anoxic by alternately applying vacuum to 0.3 bar and argon to 1.2 bar. Each bottle was supplemented NaNO_3_ to a concentration of 0.1 mM. 50 mg/l cycloheximide was added and the cell extract was incubated at 4°C for 24 h to kill predatory eukaryotic organisms. After that, the cell extract was used for inoculation.

### Medium composition

Both chemostat cultures were continuously supplied with identically prepared anoxic, sterile liquid medium containing a defined mixture of glucose, aminoacids, acetate, nitrite, and nitrate. The carbon substrates were provided in a ratio as described (Hanke et al., 2014). Briefly, ~44% of the carbon was supplied as glucose, 8% as acetate and 48% as a mixture of seven different aminoacids. The substrate concentrations were gradually increased during the first 2–7 weeks to allow gradual adaptation of the inoculated microbial community and prevent buildup of high nitrite concentrations. After this adaptation period, the concentrations in the influent medium were maintained at 20 mM for nitrite, 1 mM for nitrate and 28 mM carbon for the 25°C cultures and 37.5 C-mM for the 10°C cultures. Note that the substrate concentrations in the chemostat itself were generally in the low micromolar range as the concentrated medium was supplied to the cultures dropwise and rapidly converted by growing bacteria. Nitrite, rather than nitrate, was provided as the main electron acceptor for denitrification, because it enabled us to maintain a low (<1 mM) nitrite concentration in the culture, whereas use of nitrate resulted in higher nitrite concentrations in the culture, as explained below.

### Continuous cultivation

Continuous incubations of the sampled microbial communities lasted 100 and 250 days, for two independent sets of experiments, and were conducted in custom culture vessels (Hanke et al., 2014). The cultures were kept anoxic and the temperature was maintained at 10 and 25°C in two parallel incubations for both sets of experiments. All physical and some chemical culture conditions such as temperature, pressure, dilution rate, redox potential, and pH were automatically controlled by pumps, weight sensors underneath the culture vessels, sensors extending into the culture liquid, and finally a computer based control unit previously described (Hanke et al., 2014). Temperature was maintained with help of resistive heaters (Hanke et al., 2014) and, for the 10°C incubations, a Minichiller-NR (230 V 1~ 50/60 Hz, −20°C⋯+40°C; Peter Huber Kältemaschinenbau GmbH, Germany) coupled to a cooling finger inserted into the culture vessel. Mixing of the culture liquid was performed by the recirculation of gas from the headspace of the culture to the bottom. The culture volume was kept constant at 2.8 l by continuously feeding fresh medium and simultaneously removing spent culture liquid from the vessel. During the first 4 weeks, the dilution rate was gradually increased from 0.08 to 0.36 volume changes/day. The pH was automatically controlled at 8.0 ± 0.05 with help of NaOH and HCl. An overpressure of 13 ± 5 mbar was maintained via constantly amending the culture with 10 ml min^−1^ argon and piping spent gas though a water lock.

### Batch incubations

Batch incubations with sediment for determination of *in situ* denitrification rates were performed as described previously (Hanke et al., 2014). Briefly, fresh sediment samples were incubated in triplicates in 3-layer PA/PE tubular bags, sealed with a bag sealer, amended with 0.2 mM Na^15^NO_3_, and incubated in the dark. Batch incubations for determination of culture fermentation rates were conducted in duplicates in sterile 30 ml injection bottles (Ochs Glasgerätebau, Bovenden/Lenglern, Germany). The bottles were prefilled with 225 μl of the same sterile carbon compound stock solution used for the chemostats (400 mM-C) and 75 μl sterile HEPES stock solution of pH 8.0 (3 M initial concentration; Roth, Karlsruhe, Germany). Bottles were made anoxic by alternately applying vacuum down to 0.05 bar and Argon up to 1.5 bar, 5 times each. Fifteen milli liter culture was added to each incubation by injection. Occasionally, the culture was concentrated 2 times prior to injection by centrifuging 30 ml culture 5 min at 4700 rpm and 4°C and resuspending the pelleted cells in 15 ml anoxic artificial seawater (see above). After injecting the culture the bottles were shaken immediately to homogenize the incubation fluid. Samples taken for later analyses of fermentation products and ammonia were centrifuged 4 min at 14,500 × g and the supernatant was stored at −20°C until later analyses. Sampling started directly after shaking and was conducted 5 times at 2 h intervals. Bottles were incubated at the respective original culture incubation temperature and were shaken periodically. Batch incubations for determination of culture denitrification rate were conducted in triplicates as described above, with following differences: nitrite stock solution [sodium nitrite (Roth, Karlsruhe, Germany), 200 mM] was added to a final concentration of 0.5–0.8 mM. Carbon mixture (glucose, aminoacids, acetate, see above) stock solution (400 mM carbon) was added to 1.0–1.5 mM carbon final concentration. HEPES stock solution (3 M) was added to a final concentration of 10 mM. Culture liquid was injected without pre-concentration. Samples for later nitrite analyses were taken 7 times at 20 min intervals, centrifuged 4 min at 14,500 × g and finally the supernatant was stored at −20°C until further analyses. Production of hydrogen resulting from fermentation was measured with a Shimadzu GC-8A gas chromatograph equipped with a Molecular Sieve 5A column and a RGD2 Reduction Gas Detector (Trace Analytical, Menlo Park, CA, USA) as previously described (Pohorelic et al., 2002).

### Wet chemical analyses

Ammonia, nitrite and protein concentrations in the culture liquid were measured spectrophotometrically according to Solorzano (1969), Bendschneider and Robinson (1952), and Lowry et al. (1951), respectively. Nitrate concentrations were calculated by subtraction of nitrite concentrations (see above) from the total NOx concentration determined with an NO/NOx analyzer (2 μM detection limit; CLD 66 S, Eco Physics, München, Germany). Samples were injected into the reaction chamber connected upstream to the analyzer. At 90°C, with acid VCl_3_ (0.1 M), both NO3- and NO2-, were reduced to NO which was measured by the chemiluminescence detector. Samples for analysis of sulfide concentrations were preserved by addition of 700 uL sample to reaction tubes pre-filled with 75 uL of 50 g L-1 zinc acetate solution. Sulfide was measured spectrophotometrically according to Cline (1969). Dissolved inorganic carbon (DIC) in batch incubations was determined, after conversion to CO_2_, with a GAM400 online mass spectrometer (InProcess, Bremen, Germany). 0.5 mL liquid sample from batch incubations were injected into 5.9 mL Exetainer pre-filled with 1.5 mL 0.2 M sodium acetate buffer, pH 4. After sample injection, under pressure equilibration by passively removing gas through a second needle, samples were mixed on a vortex mixer. After 30 min, 500 uL headspace gas from the Exetainer was removed (under pressure equilibration by passively inflowing water through a second needle) and was directly injected into the mass spectrometer. Fermentation products were analyzed via high pressure liquid chromatography (HPLC) after thawing and centrifuging the samples at 14,500 × g and filtering the supernatant through a low volume (4 mm diameter) 0.45 um pore size nylon syringe filter (Chromatographie Handel Müller, Fridolfing, Germany) into 2 mL CRIMPSNAP-Vials (WICOM, Heppenheim, Germany). HPLC was conducted using the following equipment manufactured by Sykam (Eresing, Germany): S 4110 column oven, S 1100 solvent delivery system, S 8100 low pressure gradient mixer, S 5200 sample injector, and S 7510 vacuum degasser. Further equipment: ERC-7515A RI detector (40°C operation temperature; ERC, Riemerling, Germany), Sapphire UV-VIS detector (Ecom, Prague, Czech Republic), Aminex HPX-87H separating column, 300 × 7.8 mm (60°C operation temperature; Bio-Rad, Munich, Germany), and an anion neutral pre column, 4 × 20 mm (Sykam, Eresing, Germany). 5 mM H_2_SO_4_ prepared with 95–97% H_2_SO_4_ in Milli-Q water served as eluent with a flow rate of 0.6 ml/min.

### Metagenomics and DNA sequencing

Sampling for metagenomic sequencing of the 2011 cultures was conducted 97 days (25°C, sample 2011/25.1) and 101 days after inoculation (10°C, sample 2011/10.1). DNA was extracted from 50 mL of pelleted culture (Zhou et al., 1996). One micro gram of purified DNA per sample was used for the preparation of whole metagenome shotgun sequencing libraries according to the “Rapid Library Preparation Method Manual” (May 2011) for GS FLX+ Series provided by Roche (Mannheim, Germany). Libraries were sequenced on a quarter picotiterplate of GS FLX+ Titanium sequencing run. 201,559 reads (average length of 644 bases) and 192,432 reads (average length of 603 bases) were obtained for samples 2011/25.1 and 2011/10.1, respectively. Sampling for metagenomic sequencing of the 2012 cultures was conducted 35, 64, and 86 days (25°C, samples 2012/25.1, 2012/25.2, 2012/25.3, 10°C, samples 2012/10.1, 2012/10.2, 2012/10.3). DNA was extracted from 50 mL of pelleted culture (Zhou et al., 1996). 2–2.5 μg of DNA per sample were mechanically fragmented with a Nebulizer (Roche, 32 psi, 6 min in 500 μl nebulization buffer). Fragmented DNA was purified with MinElute PCR purification columns (Qiagen, Düsseldorf, Germany) and eluted in 50 μl low TE (Life Technologies, Darmstadt, Germany). The entire eluate was used to prepare barcoded PGM sequencing libraries with the Ion Xpress plus gDNA Fragment Library Preparation Kit (manual Pub. No. 4471989, Rev M, May 2013) (Life Technologies). Library insert sizes were ~200 bp. Libraries were sequenced with a Personal Genome Machine (PGM) using the chemistry of 200 bp libraries. Base calling was performed with Torrent Suite v3.2 (default settings). Samples 2012/10.1, 2012/10.2, 2012/10.3 yielded 2,045,060 reads (average length 150), 1,768,544 reads (average length 136) and 1,746,609 reads (average length 148) respectively. Samples 2012/25.1, 2012/25.2, 2012/25.3 yielded 1,370,288 reads (average length 154), 2,159,050 reads (average length 158) and 1,543,063 reads (average length 169) respectively.

### Transcriptomics and RNA sequencing

Sampling for transcriptome sequencing of the 2012 cultures was conducted 78 and 86 days after inoculation for both temperatures. Four to ten milli liter samples were centrifuged 3 min at 14,500 × g and pellets were resuspended in 300 μl Lifeguard Soil Preservation Solution (MO BIO Laboratories, Carlsbad, CA, USA) and stored at −20°C. For RNA extraction, the preservation solution was removed after centrifugation and pellets were resuspended in 1 ml TRI Reagent solution (Applied Biosystems, Darmstadt, Germany). The suspension was transferred to a bead beater tube containing 0.25 mL sterile 0.1 mm glass beads for bead beating at 6.5 m/s for 45 s. After incubation at room temperature (RT) for 5 min, the tube was centrifuged for 5 min at 12,000 × g and 4°C and the supernatant was transferred to a fresh tube. Two hundred micro liter chloroform were added followed by vigorous shaking by hand for 15 s, incubation at RT for 10 min, and centrifugation at 12,000 × g, 4°C for 15 min. The upper phase was transferred to a fresh tube, 500 μl ice-cold isopropanol were added and the tube was inverted several times, followed by incubation on ice for 30 min for RNA precipitation. After centrifugation at 20,000 × g, 4°C for 25 min, the pellet was washed with 1 mL ice-cold ethanol three times (10 min centrifugation at 20,000 × g, 4°C between washing steps) and air dried at RT for ~10 min. The pellet was resuspended in sterile TE buffer (pH 8.0) and incubated on ice for ~30 min for complete dissolution of RNA. The extracted RNA was treated with DNase (Promega, Mannheim, Germany) and purified with Rneasy MinElute spin columns (Qiagen, Düsseldorf, Germany). Before library preparation, rRNA was depleted from purified RNA (3–5 μg) using the Ribo-Zero rRNA removal kit (Bacteria, Epicentre, Madison, WI, USA). Libraries were prepared with the Ion Total RNA-Seq Kit v2 (Life Technologies) following the protocol for whole transcriptome library preparation. The generated cDNA libraries were sequenced with a Personal Genome Machine (PGM), with chemistry for 200 bp libraries. Base calling was performed with Torrent Suite v3.2 (default settings).

### *In silico* procedures

Sequencing reads were quality filtered, trimmed, detagged and assembled with the GS De Novo assembler 2.8 (454 Life Sciences, Branford, CT, USA) using the default settings for genomic DNA. On average, 85% of the reads were assembled into contigs. Assembled contigs were binned with MetaWatt 2.5 (Strous et al., 2012). Overall, 15 bins, each with a consistent phylogentic profile, were identified. Per contig sequencing coverage was estimated by mapping the reads to the assembled contigs with bowtie2 (Langmead and Salzberg, 2012) and coverage and bin size were used to estimate the abundance of each binned population. For each bin, genome completeness was estimated by representation of 139 conserved single copy genes (Campbell et al., 2013). Genome completeness was also assessed by counting the number of encoded tRNA genes (Laslet and Canback, 2004). The contigs constituting each bin were annotated with Prokka (Seemann, 2014). 16S rDNA gene sequences were obtained by searching the assembled contigs with custom Hidden Markov Models (Eddy, 2011) trained with representative sequences from the SILVA database (Quast et al., 2013) and, independently, by iterative read mapping with EMIRGE (Miller et al., 2011). Multiple sequence alignment of 16S rDNA gene sequences was performed with MAFFT (Katoh et al., 2002; options –maxiterate 1000 –localpair) and a phylogenetic tree was calculated by approximate maximum likelihood with FastTree2 (Price et al., 2010) after application of a 25% conservation filter. Key functional genes (*nirS, nirK, nosZ, pflB*) were detected with custom Hidden Markov Models, one for each gene, calculated from multiple sequence alignments of representative genes from reference genomes. Prior to detection, all contigs were translated in six frames DNA regions potentially encoding one of these functional genes were analyzed further with BLASTx against the refseq database. Reads obtained from reverse transcribed extracted RNA were mapped against the assembled contigs and per-gene transcriptional activities were calculated. These activities were normalized for gene length and for the average transcriptional activity for the bin as a whole. This way, the average transcriptional activity of genes belonging to any given bin was equal to 1. A value above one indicated above average activity and a value below 1 indicated below average activity.

### Data submission

The generated sequencing reads are accessible via the NCBI resource platform http://www.ncbi.nlm.nih.gov/bioproject under PRJNA255460. The individual accession numbers for the DNA samples are: SAMN02919310, SAMN02919311, SAMN02919312, SAMN02919313, SAMN02919314, SAMN02919315, SAMN02919316, SAMN02919317. And for the RNA samples: SAMN04157987, SAMN04157988, SAMN04157989, SAMN04157990.

## Results and discussion

A tidal flat in the German Bight was sampled in the month of March of two subsequent years (2011 and 2012). At the time of sampling, the water temperatures were 3 and 8°C respectively, compared to a mean (1948–2012) temperature of 4°C in March. Mean temperatures peaked at 16°C in August, but temperatures of sediments exposed to direct sunlight as high as 32°C have been recorded (CWSS, 2014)^1^. Seasonal potential sediment denitrification rates were measured between October 2009 and July 2010. Denitrification rates in March were ~0.8 μmol N^2^ L^−1^ day^−1^ independent of incubation temperature (10–30°C). Potential denitrification rates were highest in October. In October, rates depended on temperature and increased from (2.2 ± 0.6) to (6.1 ± 0.7) μmol N^2^ L^−1^ day^−1^ between 10 and 30°C.

Bacteria extracted from sediment samples in March 2011 were incubated in two chemostats, one at 10 and one at 25°C. This procedure was repeated in March 2012. Each chemostat was maintained anoxically for at least 100 days. The chemostats were supplied with medium containing nitrite as the main electron acceptor. Some nitrate was provided in addition to nitrite, but only a relatively small amount (1 mM). In response to nitrate, the cells extracted from the sediment rapidly converted the provided nitrate to nitrite, leading to high (>1 mM), potentially toxic, transient nitrite concentrations in the cultures, if the influent nitrate concentration was too high. During the first 4 weeks of the incubation, the nitrite concentration in the medium was gradually increased from 5 to 20 mM. The influent concentration of nitrite was adjusted so that it was always (almost) completely consumed and the actual nitrite concentration in the chemostat cultures remained below 1 mM. During the first 4 weeks, the dilution rates were gradually increased, up to 0.36 volume changes/day.

The medium also contained a mixture of carbon compounds (glucose, acetate, and seven different aminoacids) in a ratio that mimicked the composition of decaying biomass—the main carbon and energy source *in situ*—in terms of its monomers (Hanke et al., 2014).

Initially, the 10 and 25°C chemostats received nitrite and organic carbon in the same ratio (1.25 C-mol/N-mol in 2011 and 0.9 C-mol/N-mol in 2012). At 25°C, nitrite and nitrate was completely (detection limit 10 μM) converted. With regard to the carbon compounds, these were at least partially fermented, as fermentation products (acetate, formate, succinate, up to 1.8 mM total) were detected in the spent culture medium. Although acetate was already present in the culture medium, formate and succinate were not, so these at least must have resulted from microbial fermentation. This may indicate that the carbon provided was more than sufficient for complete denitrification and the remainder was fermented. In any case, nitrite and nitrate were the controlling (limiting) substrates for the denitrifying populations enriched in the chemostat at 25°C.

Fermentation products were also detected in the spent medium of the 10°C cultures. However, at 10°C the supplied nitrite was often not consumed completely, in 2011 as well as and 2012 cultures. Whenever the nitrite concentration in the chemostat increased to >1 mM, the concentration of organic compounds in the influent medium was increased or the dilution rate was decreased. Both interventions temporarily led to complete consumption of nitrite. However, within a few days, nitrite accumulated once more and this could only be reversed by further increasing the concentration of organic compounds or further decreasing the dilution rate. Eventually, the dilution rate was 0.08 volume changes/day and the C:N ratio was 1.8 C-mol/N-mol. Fermentation products (acetate, butyrate, propionate, succinate, lactate, up to 3.1 mM total) were detected in the culture medium.

The protein concentration measured in the 2011 cultures was stable at (0.14 ± 0.05 g/L) at 25°C and (0.12 ± 0.02 g/L) at 10°C. In 2012, the protein concentration was stable at (0.095 ± 0.01 g/L) at 25°C and (0.1 ± 0.03 g/L) at 10°C. Note that high concentrations of the carbon sources were provided to the 10°C culture. These protein concentrations were consistent with concentrations of ammonium in the spent culture medium. For example, for the 2011, 10°C culture, the influent medium contained 5.3 mM of organic nitrogen, and the ammonium concentration in the spent medium was 2.5 mM. The difference must have been assimilated or could have resulted from dissimilatory nitrate reduction to ammonia (DNRA). In the absence of DNRA, with a biomass C:N ratio of 0.2, this difference would correspond to a protein concentration of 0.15 g/L. A concentration of 0.12 g/L was measured. These protein concentrations indicated that at 25°C, ~0.4 C-mol carbon substrate was assimilated per C-mol converted, both in 2011 and in 2012. Previously, growth yield values of 0.3 C-mol assimilated/C-mol converted were reported for the model denitrifiers *Paracoccus denitrificans* and *Pseudomonas stutzeri* growing on acetate (Strohm et al., 2007). The slightly higher growth yield observed at 25°C should still be considered consistent with these values, because the chemostats were, in addition to acetate, also provided with glucose and aminoacids.

Interestingly, the growth yields observed at 10°C (0.2–0.3 C-mol assimilated/C-mol converted) were equal or lower than those of the model organisms, despite the availability of excellent substrates such as glucose and aminoacids.

**Figure 1:**
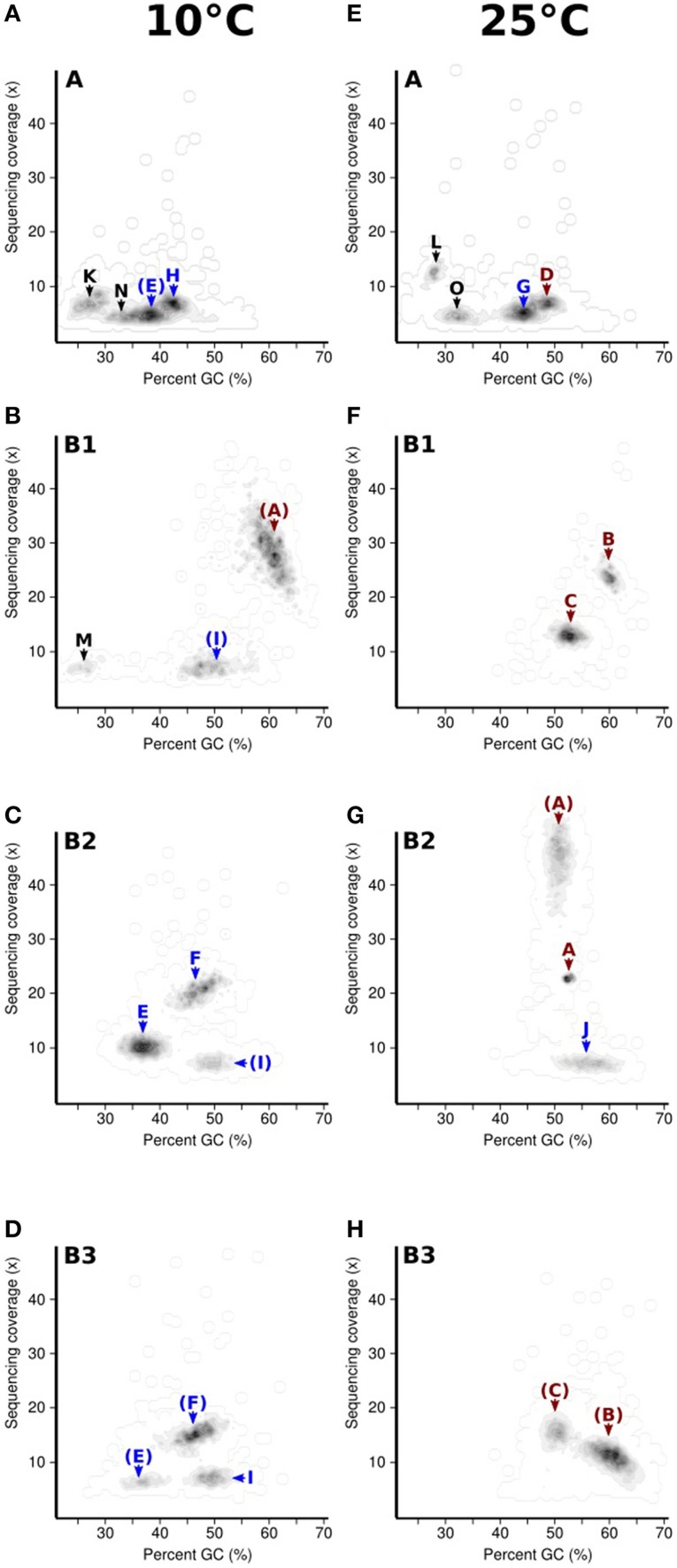
**Distribution of assembled contigs for the eight metagenomes sampled from the chemostat (2011 cultures 10-A, A and 25-A, E, 2012 cultures 10-B1-3, B-D, 25-B1-3, F-H) with respect to GC-content and sequencing coverage**. Blobs of contigs with similar GC-content and coverage roughly correspond to bins obtained by tetranucleotide binning. For each blob, the corresponding bin identifier (A-O) is indicated. Bins affiliated to Alphaproteobacteria and Gammaproteobacteria are shown in red and blue respectively. Parenthesized bin identifiers indicate the bin was detected but that the quality of the bin (N50 contig length) was lower than in another sample.

**Figure 2:**
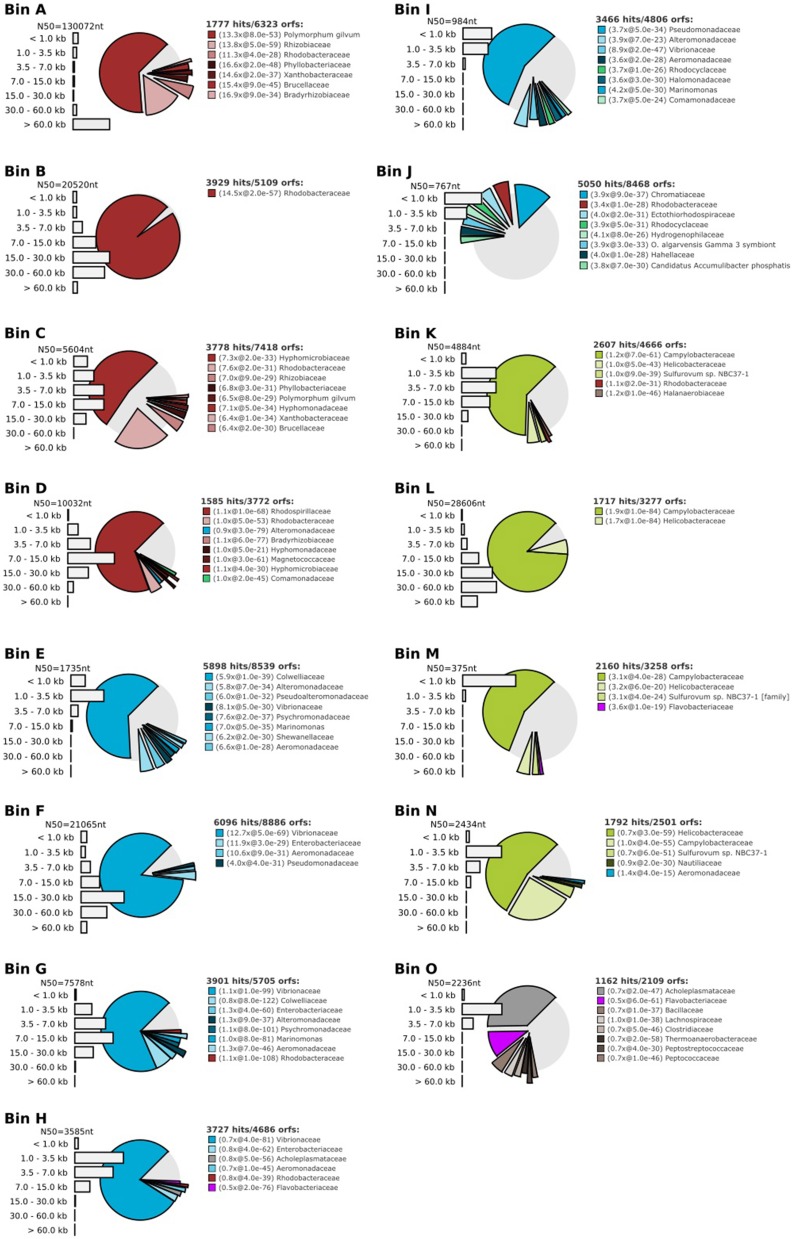
**Taxonomic profile for each bin A-O, demonstrating binning effectiveness**. These profiles were created by fragmenting the contigs belonging to each bin into 500 base pair fragments and blasting each fragment to a database containing genomes of ~1000 reference bacteria. The bar graphs show the length distribution of the contigs making up the bin.

**Figure 3:**
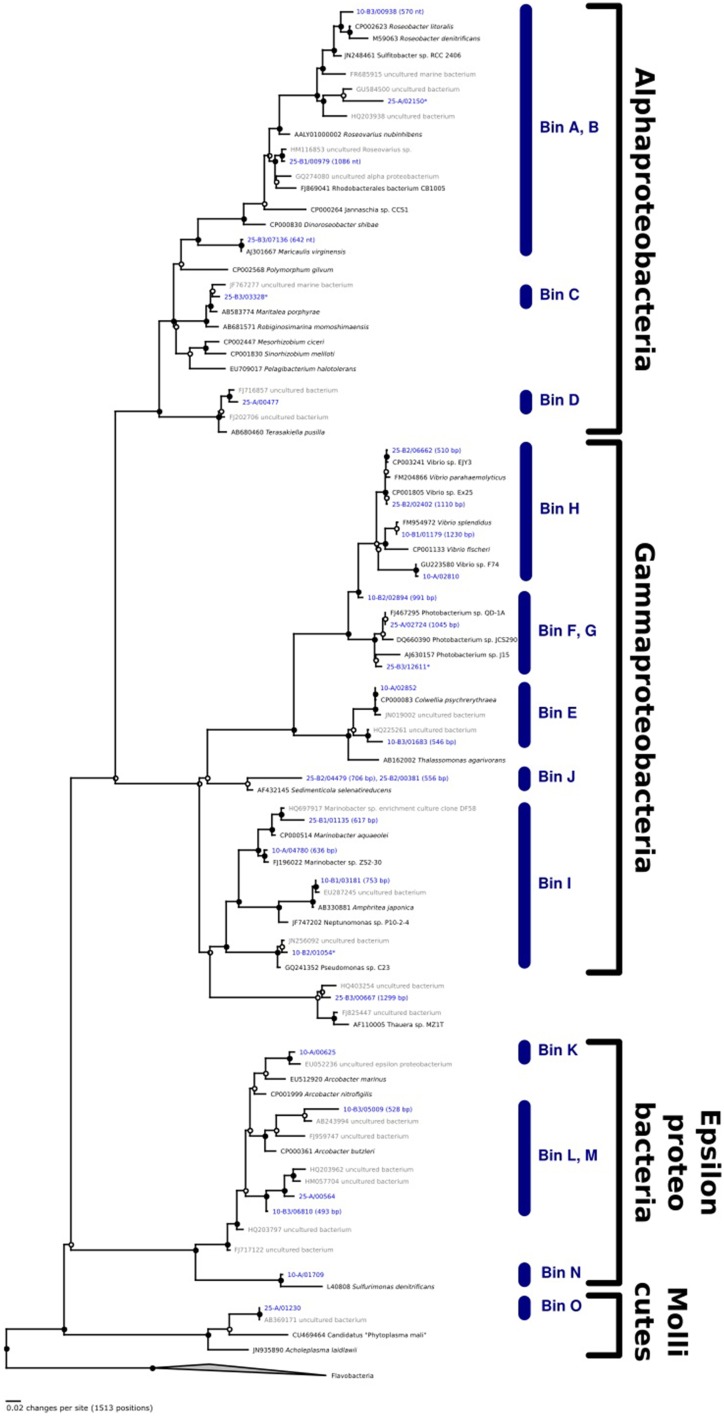
**Phylogenetic analysis of 16S rDNA genes extracted from the assembled contigs**. Lengths (in base pair) are indicated for those genes that were not full length. Those genes that were recovered independently by iterative read mapping are indicated with an asterix. The bin corresponding to each 16S rDNA gene is indicated. For some bins (e.g., A,B) the 16S rDNA gene sequences could not be assigned unambiguously.

**Figure 4:**
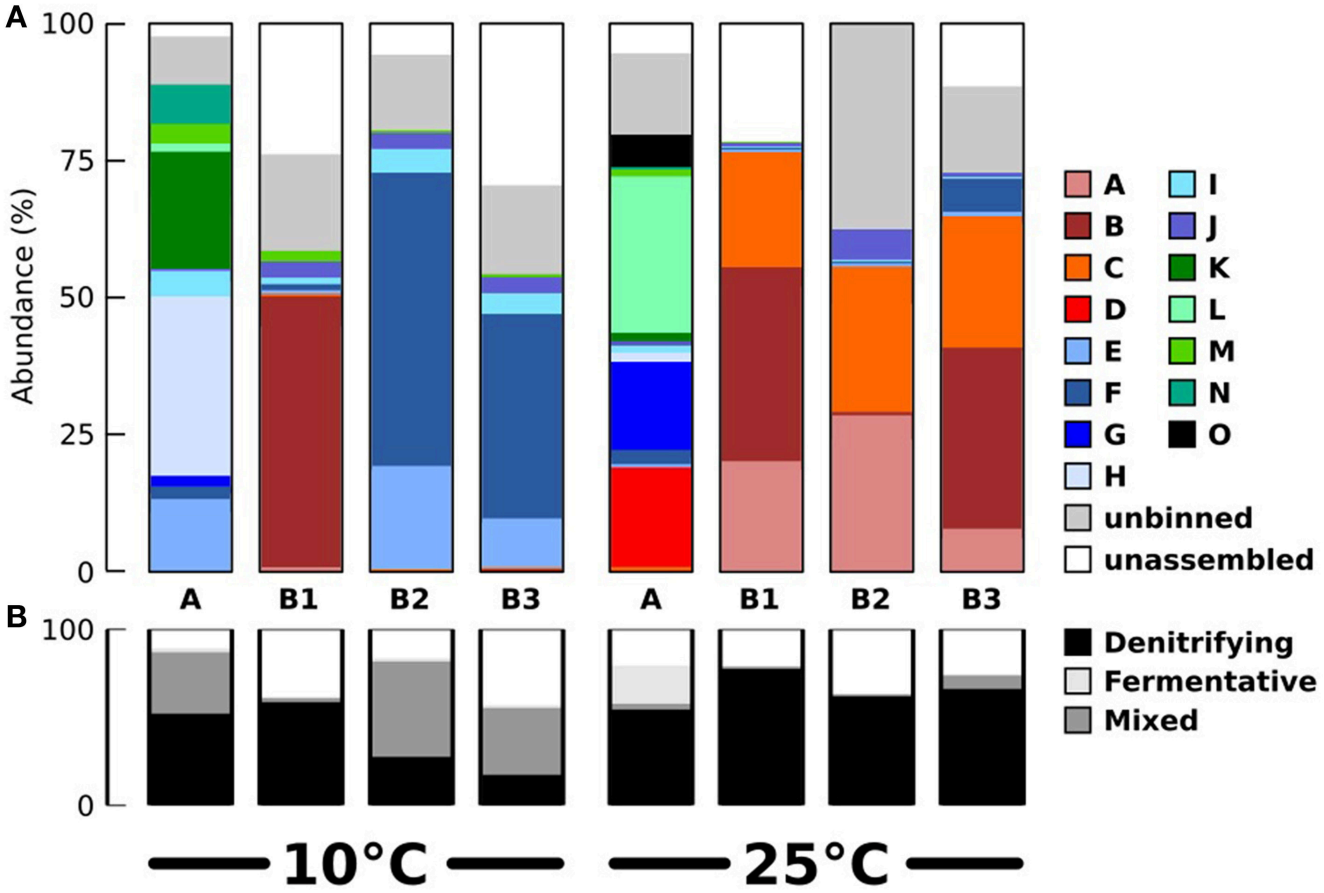
**Estimated abundances of the binned populations and their contributions to denitrification and fermentation**. **(A)** Population abundances in the eight samples, based on mapping of sequencing reads to the assembled contigs of each bin. Populations affiliated with Alpha-, Gamma-, and Epsilonproteobacteria are shown in red/orange, blue, and green colors respectively. **(B)** Abundance of populations contributing to denitrification, fermentation and both processes, based on detection of functional genes.

Seeking qualitative explanations for these observations at the molecular level, we performed shotgun metagenomic sequencing of DNA extracted from the chemostat cultures. One DNA sample was sequenced for each 2011 cultures (day 85, samples 10-A and 25-A) and three DNA samples were sequenced from the 2012 cultures (days 65, 75, 85, samples 10-B1, 10-B2, 10-B3, 25-B1, 25-B2, and 25-B3). Sequencing reads were quality filtered and trimmed, and assembled into contigs. The contigs were binned into provisional whole genome sequences (WGS). Figure 1 shows each of these WGS as blobs on a percent-GC vs. sequencing coverage plot and Figure 2 shows the phylogenetic distribution of blast hits for the assembled contigs making up each WGS. Table 1 shows the basic properties of the provisional WGS. Figure 3 shows the phylogeny of the 16S rDNA sequences extracted from the assembled contigs and/or assembled independently by iterative read mapping with Emirge (Miller et al., 2011). Both assembly and binning of ribosomal gene sequences can be challenging and therefore, assignment of 16S rDNA genes to bin was based on co-occurrence patterns and phylogeny. The phylogeny of the 16S sequences corresponded well to the phylogenetic profiles of the binned contigs but some bins were associated with multiple, non-identical 16S rDNA sequences, indicating that multiple, related populations may have been cross-assembled and/or cross binned. See for example a double coverage maximum for bin A in sample 25-B2 in Figure 1 and the presence of multiple versions of conserved single copy genes (bins A, G, H, K) shown in Table 1. The abundances of the 15 populations, each associated with a provisional WGS (Table 1), in all samples was estimated by mapping all sequencing reads to the contigs of each WGS (Figure 4A). This approach was more sensitive than de-novo assembly. In many samples the abundance of some of the 15 populations was too low to enable assembly, but the abundance of these populations could still be detected by read mapping. For example, in sample 25-B3, bin J (affiated with *Chromatiales*) did not assemble effectively (Figure 1, Table 1) but was still detected by read mapping (Figure 4A). Previously, it was shown for similar populations that estimates obtained with this approach were in excellent agreement with results from fluorescence *in situ* hybridization (FISH, Hanke et al., 2014).

**Table 1:** **Properties of the 15 bins obtained by tetranucleotide binning of eight metagenomes sampled from the chemostat cultures (see also Figures 1, 2)**.

**Bin**	**A**	**B**	**C**	**D**	**E**	**F**	**G**	**H**
**Affiliation**	**Rhodo-bacterales**	**Rhodo-bacterales**	**Maritalea**	**Terasakiella**	**Colwellia**	**Photobacterium**	**Photobacterium**	**Vibrio**
Size (kb)	5.85	3.34	3.82	3.37	4.13	5.77	3.74	5.21
Number of contigs (#)	2552	558	2076	523	3520	1147	1392	1013
N50 contig length (kb)	71.8	20.5	5.6	10.0	1.7	21	3.6	7.6
GC content (%)	51.6	59.3	52.0	47.7	36.2	47.3	43.4	41.7
Number of CSCGs^*^ (#)	235	113	131	111	111	134	158	163
Number of tRNAs (#)	53	40	32	34	25	54	66	52
Sample/Bin	25-B2/M0	25-B1/L0	25-B1/FD	25-A/L1	10-B2/M4	10-B2/L0	25-A/L2	10-A/L0
**Bin**	**I**	**J**	**K**	**L**	**M**	**N**	**O**	
**Affiliation**	**Pseudo-monas**	**Chromatiales**	**Arcobacter**	**Arcobacter**	**Arcobacter**	**Sulfuri-monas**	**Achole-plasmatales**	
Size (kb)	2.38	3.30	3.45	2.92	0.96	1.61	1.41	
Number of contigs (#)	3141	5349	1095	206	2640	762	704	
N50 contig length (kb)	0.97	0.77	4.9	28.6	0.38	2.4	2.2	
GC content (%)	48.8	57.0	26.9	27.6	29.0	32.5	32	
Number of CSCGs^*^ (#)	118	80	170	113	55	95	96	
Number of tRNAs (#)	22	29	40	33	10	23	13	
Metawatt bin	10-B3/L3	25-B2/H6	10-A/L1	25-A/L0	10-B1/L5	10-A/L2	25-A/L4	

* *Number of Conserved Single Copy Genes detected (out of a set of 139). Numbers higher than 139 indicate the presence of DNA originating from more than a single population in the bin. Numbers lower than 139 indicate the provisional genome sequence associated with the bin may be incomplete*.

All abundant populations were affiliated to Proteobacteria except for one minor population (detected in sample 25-A) affiliated with Mollicutes. Most abundant populations in the 25°C chemostats were affiliated with Alphaproteobacteria. Most abundant populations in the 10°C chemostats were affiliated with Gammaproteobacteria, except for the presence of the population associated with bin B in sample 10-B1. Epsilonproteobacteria were also frequently present at 10°C. We tested publicly available amplicon sequencing data (Fuhrman et al., 2008; Sharp et al., 2014) whether abundance of Gammaproteobacteria and Alphaproteobacteria generally correlated positively with low and high temperatures respectively, but this was not the case. Therefore, this trend appears to be coincidental or at most specific to the sampled tidal flat and/or the subsequent chemostat selection. At 10°C, only a single known psychrophile was detected (bin E, related to *Colwellia psychrerythraea*).

The microbial community composition, as inferred from metagenomic sequencing, in the 2012 cultures was fairly stable, at least between days 65 and 85, with one exception, the abundance of the population associated with bin B in sample 10-B1. However, the community compositions in the 2011 cultures were very different from those observed in the 2012 cultures (Figure 4A). This might have been caused by differences in the inoculum communities sampled from the tidal flat in different years, or by stochastic factors during chemostat selection. The populations dominating the 2011 cultures were still detectable in the 2012 cultures, but sometimes at very low numbers. This was for example the case for the population most closely related to *Terasakiella pusilla*, bin D).

Despite the differences between the 2011 and 2012 microbial communities, the outcome of selection at 10 and 25°C was the same at the functional level. Both in 2011 and in 2012, denitrification was stable at 25°C. At 10°C, complete denitrification was only maintained with difficulty and depended on manual interventions in the form of a gradually lowered dilution rate and gradually increased supply of organic carbon. Apparently, independent of microbial community composition, denitrification was negatively affected by a lower temperature.

To further investigate this effect, open reading frames were predicted and genes were annotated for each of the 15 provisional WGS (Supplementary Material). Transcriptional activities of each gene were determined for the 2012 cultures by sequencing reversed transcribed RNA extracted from the 10 and 25°C cultures on days 70 and 80. While analyzing the automated annotations, we noticed, that because of presumably artefactual frame shifts within genes (ion torrent technology is known to have difficulty in sequencing mono-nucleotide strings) combined with relatively short contigs for some bins, some open reading frames were not predicted correctly. Therefore, we performed a more in depth analysis focusing on the key functional genes involved in denitrification (*nirS*, cytochrome cd1 nitrite reductase, *nirK*, copper dependent nitrite reductase, *nosZ*, nitrous oxide reductase) and fermentation (*pflB*, pyruvate formate lyase). *PflB* was the only gene that could be unambiguously assigned to fermentation, because, for example, genes involved in acetate production are bi-directional and could also be used for acetate uptake. Pyruvate formate lyase converts pyruvate to formate and acetyl-CoA. The latter is subsequently phosphorylated and converted to acetate and ATP by substrate level phosphorylation. These four genes were detected by searching six-frame translations of all contigs with HMM profiles. The identity of the identified genes was subsequently validated with a blastx search against the refseq database. For each gene, the transcriptional activity was inferred from the sequenced transcriptomes (see Table 2).

**Table 2:** **Key functional genes involved in denitrification (NirK, NirS, NosZ, encoding Copper-type nitrite reductase, cytochrome cd1 nitrite reductase and nitrous oxide reductase respectively) and fermentation (pflB, encoding pyruvate formate lyase) detected on binned contigs**.

**Contig**	**Gene**	**Contig length**	**Gene position**	**Strand**	**Affiliation**	**Activity**	**Bin**
25-B2/contig00017	*nirK*	71,778	45,446–46,495	−	*Maritalea*	1.5	A
25-B1/contig00217	*nirK*	9174	2,768–3662	−	*Maritalea*	1.7	C
25-A/contig00033	*nirS*	30,072	25,343–26,816	−	*Thalassospira*	0.1	D
25-A/contig00158	*nirS*	12,152	6680–8231	−	*Thioalkalivibrio*	3.2	D
10-B2/contig00974	*nirS*	2242	904–2232	−	*Colwellia*	2.6	E
25-B2/contig00523	*nirS*	2510	980–2228	−	*Sedimenticola*	0.0	J
25-B2/contig01160	*nirS*	1675	4–1195	−	*Sedimenticola*	3.8	J
10-A/contig00157	*nirS*	10,634	2400–3807	+	*Nitratiruptor*	3.9	K
25-A/contig00002	*nirS*	107,659	45,626–47,102	−	*Methylomonas*	-	L
25-A/contig00024	*nirS*	33,188	22,075–24,286	−	*Sulfurimonas*	-	L
10-B1/contig19936	*nirS*	313	1–313	+	*Sulfurovum*	-	M
25-B2/contig01207	*nosZ*	1639	2–1634	+	*Maritalea*	31	A
25-B2/contig03862	*nosZ*	794	6–792	−	*Maritalea*	7.4	A
25-B2/contig05570	*nosZ*	586	14–577	+	*Alcaligenes*	25	A
25-B1/contig00005	*nosZ*	50,905	24,280–26,089	−	*Ruegeria*	1.5	B
25-A/contig00045	*nosZ*	25,838	2465–4382	−	*Azospira*	1.9	D
10-B2/contig01391	*nosZ*	1717	554–1714	−	*Colwellia*	4.9	E
10-B2/contig00170	*nosZ*	12,996	5–1668	−	*Photobacterium*	4.4	F
10-A/contig00784	*nosZ*	4544	272–2114	−	*Photobacterium*	0.8	H
10-B3/contig01180	*nosZ*	1384	30–1341	+	*Pseudomonas*	1.4	I
10-B3-contig01396	*nosZ*	1256	25-385	+	*Pseudomonas*	2.7	I
25-B2/contig00886	*nosZ*	1,931	919–1921	−	*Sedimenticola*	3.8	J
25-B2/contig00727	*nosZ*	2135	8–889	−	*Sedimenticola*	3.7	J
10-A/contig00156	*nosZ*	10,642	5801–7757	−	*Campylobacter*	3.4	K
25-A/contig00195	*nosZ*	10,710	8678–10,643	−	*Campylobacter*	0.6	L
10-A/contig04078	*nosZ*	1065	7–1063	−	*Wolinella*	-	N
10-B2/contig00028	*pflB*	33,743	4060–6813	+	*Vibrio*	0.04	F
10-B2/contig00038	*pflB*	31,309	8080–9932	−	*Photobacterium*	1.4	F
10-B2/contig00238	*pflB*	9510	8098–9499	+	*Vibrio*	0.1	F
25-A/contig00439	*pflB*	5996	54–1652	+	*Enterobacter*	-	G
25-A/contig01759	*pflB*	1841	46–1834	−	*Vibrio*	1.2	G
25-A/contig02479	*pflB*	1235	6–1185	−	*Vibrio*	-	G
10-A/contig00058	*pflB*	15,024	9499–11,539	+	*Vibrio*	1.8	H
10-A/contig00738	*pflB*	4717	1138–3426	+	*Tolumonas*	-	H
10-A/contig01764	*pflB*	2564	427–2552	+	*Vibrio*	-	H
25-A/contig01805	*pflB*	1800	9–1793	−	*Acholeplasma*	-	O

Gene activity was inferred from transcriptomic analysis and normalized for both gene length and the average activity of the bin as a whole. A normalized value of 1.0 indicates average gene activity. Transcriptomes were only performed for the 2012 culture. If no transcripts were detected for those populations present in the 2011 cultures, this is indicated with “-.”

All four detected Alphaproteobacteria appeared to be involved in denitrification. Two populations encoded and transcribed both nitrite reductase and nitrous oxide reductase. Two populations encoded only one of the investigated denitrification genes. All Gammaproteobacteria encoded and transcribed *nosZ*, except for a population affiliated with *Photobacterium* (bin G). A population affiliated with *Colwellia* (bin E) and one affiliated to *Pseudomonas* (bin I) also encoded and transcribed *nirS*. All Epsilonproteobacteria appeared to be involved in denitrification. Two Epsilonproteobacterial populations encoded both nitrite reductase (*nirS*) and nitrous oxide reductase. Two populations encoded only one of the investigated denitrification genes. Pyruvate formate lyase was present and active in three populations affiliated with *Photobacterium*/*Vibrio* (bins F, G, H) and in the population affiliated with Mollicutes (bin O).

**Figure 5:**
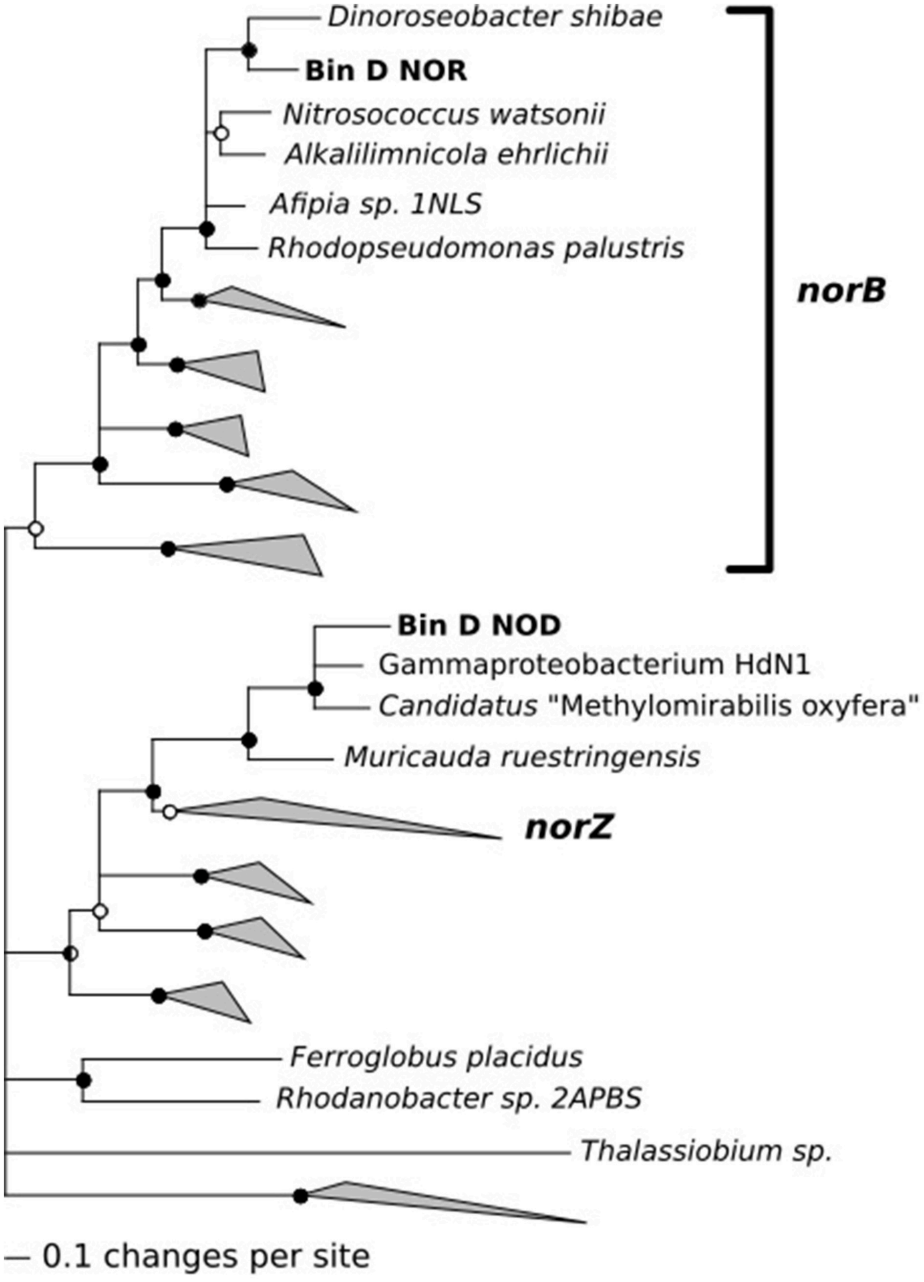
**Phylogenetic analysis of the nitric oxide reductase (NorB) and the putative nitric oxide dismutase (Nod) encoded on the contigs of bin D**.

While analyzing nitric oxide reductase genes (*norB/norZ*), we made one unexpected observation: The population related to *Terasakiella pusilla* (bin D) appeared to encode a putative nitric oxide dismutase (*nod*) that may catalyze the dismutation of two molecules of nitric oxide into N_2_ and O_2_ (Ettwig et al., 2010, 2012). Phylogenetically, the predicted gene product was closely related to putative nod genes of Candidatus “*Methylomirabilis oxyfera*” and Gammaproteobacterium strain HdN1 (Zedelius et al., 2011, see Figure 5). Both these bacteria have been proposed to convert nitric oxide into oxygen, which is used in the subsequent mono-oxygenation of methane or long chain alkanes respectively. The Nod protein encoded in bin D also contained the same modifications to the active site that set putative *nod* gene products apart from typical quinone dependent nitric oxide reductases. The 16S rDNA gene sequence associated with bin D was found to be identical to that of a bacterium previously isolated from a North Sea oil field exposed to nitrate (Bødtker et al., 2009). No alkanes or methane were provided to the chemostats in the present study and bin D also encoded a typical NorB nitric oxide reductase. The population associated with bin D was not abundant in the 2012 cultures (for which transcriptomes were available), but transcription of its *norB* gene was still detectable. This was not the case for the *nod*. Apparently, the population is well adapted to oil fields but is still sufficiently versatile to enable selection in the present study, using glucose, aminoacids and/or acetate as potential substrates.

Based on the detection of the functional genes involved in denitrification and fermentation, the 15 populations for which provisional WGS were available were classified as denitrifiers (bins A–E, I–N), fermentative (bin O) and combined denitrifiers/fermentative (bins F–H). Figure 4B shows the aggregated abundances of these three functional groups in the different samples. This analysis shows that fermentative/denitrification co-metabolism was prevalent mainly at 10°C and that the relative abundance of specialized fermentative and denitrifying populations was lower at 10°C compared to 25°C. It should be noted here that because only a single fermentative pathway (via pyruvate/formate lyase) was assessed, it cannot be excluded that these “specialized” denitrifying populations related to *Rhodobacteraceae* might still have engaged in other forms of fermentation (e.g., fermentation of aminoacids).

Combined respiratory and fermentative metabolism is well known for many aerobic bacteria and eukaryotes and is sometimes referred to as the Crabtree effect (Crabtree, 1928). However, compared to the aerobic case, the microbial ecology of the combined fermentative/denitrification co-metabolism is more complex, because only a single step of the denitrification pathway is performed (nitrous oxide reduction) and these populations still depend on a different population that performs the upstream steps of denitrification. This may be a general pattern, as nitrous oxide is present more often in genomes of *Vibrionales*, as well as in facultatively fermentative Flavobacteria (Jones et al., 2012).

Our observations may lead us to conclude that at 25°C denitrifying bacteria generally out-competed fermentative bacteria for the provided substrates or engaged in stable syntrophic interactions with upstream fermenters. Somehow, at 10°C, these types of denitrifying bacteria (*Rhodobacteraceae*) were less successful, and fermentative, nitrous oxide reducing bacteria became prevalent. Because the latter would still produce fermentation products that could in theory be used as substrates by *Rhodobacteraceae*, it remains unknown why the denitrifiers co-enriched at 10°C (related to *C. psychrerythacaea, Pseudomonas*, and *Arcobacter*) did not manage to sustain denitrification as a stable ecosystem function. At 10°C, these denitrifying bacteria must have been affected by other factors that escaped our analyses, such as antagonistic effects between bacterial populations or viral predation.

Overall, metagenomic investigation of denitrifying and fermentative microbial communities selected in laboratory chemostats revealed two different ecological regimes acting on these two processes and resulting in poor performance of denitrification at 10°C. Despite the fact that different populations were selected in two replicate experiments performed in two subsequent years, the functional outcome was the same. Although the use of chemostats enables more or less stable conditions for prolonged periods of time, at low substrate concentrations as observed in the environment, it remains to be seen whether the two different regimes also operate on natural ecosystems, such as the tidal sediments used as the inoculum in the present study. However, this study clearly showed that the functioning of denitrifying bacteria is not only affected by oxygen, but also by organic carbon availability. Just like oxygen was previously shown to control denitrification rates, with lower temperatures resulting in decreased denitrification rates (Veraart et al., 2011), the present study shows that ecological interactions between bacteria performing denitrification, fermentation or both may also compromise denitrification rates at low temperatures.

Both next generation sequencing itself and computational tools for the analysis of metagenomes are showing amazing technological progress. Would this study be repeated today, the combination of more, longer and more accurate sequencing reads, and better assembly, binning and annotation tools would enable extraction of more and higher quality provisional whole genome sequencing, resulting in more accurate inferences. The present study only enabled a first glimpse of the causes underlying the experimental observations, and showed that presently unknown ecological interactions critically affected the investigated processes. Further progress will require better resolved time series and a holistic approach that not only addresses the processes themselves but also takes into account these other, less investigated ecological interactions.

## Author contributions

AH performed all experiments with help from JB and TH. HT performed next generation DNA sequencing, read quality control and assembly. MS performed binning, annotation, and read mapping. CS analyzed abundance of Alpha- and Gammaproteobacteria in environmental datasets. The manuscript was written by AH and MS with input from all other co-authors.

## Funding

This research was funded by an ERC starting grant to MS (MASEM, 242635), the German Federal State North Rhine-Westphalia, the Max-Planck-Gesellschaft and the Campus Alberta Innovation Program.

### Conflict of interest statement

The authors declare that the research was conducted in the absence of any commercial or financial relationships that could be construed as a potential conflict of interest.

## Footnotes

^1^CWSS (2014). Available online at: http://www.waddensea-secretariat.org/
